# Anger Responses in Adolescents: Relationship with Punishment and Reward Sensitivity

**DOI:** 10.1007/s10578-021-01191-w

**Published:** 2021-06-07

**Authors:** L. J. Kreuze, P. J. de Jong, E. C. Bennik, M. H. Nauta

**Affiliations:** Department of Clinical Psychology and Experimental Psychopathology, University of Groningen, The Netherlands Grote Kruisstraat 2/1, 9712 TS Groningen, The Netherlands

**Keywords:** Anger, Adolescents, Punishment sensitivity, Reward sensitivity, Children’s inventory of anger

## Abstract

A substantial proportion of youth with anxiety disorders shows comorbid behavioral (anger) problems. Such comorbid profile is associated with low treatment effectiveness and negative (longterm) outcomes. This study was therefore designed to examine trait factors that may promote anger responding in adolescents. By presenting participants (N = 158, mean age = 15.7, 56% female) with a series of common anger-eliciting situations, we tested whether high reward sensitivity would be associated with anger via perceived non-reward, and high punishment sensitivity via perceived threat. In line with the hypotheses, an indirect effect of reward sensitivity on anger was found via perceived non-reward, and an indirect effect of punishment sensitivity on anger via perceived threat. The latter association also had an indirect effect via perceived non-reward. High punishment and reward sensitivity may thus set adolescents at risk for developing (comorbid) anger problems via heightened threat and non-reward perceptions.

**Supplementary Information:**

The online version contains supplementary material available at 10.1007/s10578-021-01191-w.

## Introduction

Anxiety disorders are among the most prevalent disorders in childhood [1]. Intriguingly, about 20% of children and young people (CYP) with an anxiety disorder also meet criteria for a disruptive behavioral disorder, characterized by anger and oppositional behaviors [2–4]. Importantly, it has been found that children with anxiety and comorbid anger problems profit less from current treatment approaches [5, 6], have greater impairment in daily life [7–10], and are at increased risk for continued mental health problems in adulthood [6, 11]. Insight in the underlying processes of this comorbidity may provide clues that can help improve these children’s condition.

Two important traits that have often been linked to symptoms of anxiety and behavioral disorders are sensitivity to punishment (PS) and sensitivity to reward (RS). Punishment and reward sensitivity stem from the reinforcement sensitivity theory [12–15]. They are presumed to represent orthogonal dimensions that can vary independently in strength indicating that all combination of (relatively) high and (relatively) low PS and RS may be evident in a particular population [16]. Individuals at the far poles of punishment and/or the reward sensitivity dimensions are expected to have an increased risk for developing mental health problems [17], which might especially becoming evident during periods with increasing demands, such as adolescence and young adulthood [18]. This study therefore examined whether high punishment and/or high reward sensitivity may also help explain the co-occurrence of oppositional problems in youth with anxiety disorders. More specifically, this study was designed to test two candidate pathways that may help explain these anger responses in adolescents.

One pathway may stem from heightened punishment sensitivity. There is ample evidence indicating that children and adults with anxiety symptoms and anxiety disorders are characterized by high punishment sensitivity [19–22]. People with high punishment sensitivity have a relatively strong inclination to interpret ambiguous situations in a threatening way [12, 23]. Consistent with the view that such a threat bias may help explain the relationship between punishment sensitivity and anxiety, there is evidence that the relationship between punishment sensitivity and anxiety is mediated by cognitive biases for negative and threatening information [24]. Importantly, perceived threat may not only elicit anxiety, but also defensive anger. This is in line with the social information processing theory, which suggests that adolescents with behavioral problems interpret social information in ways that increases their likelihood to become angry [25]. Emotions have adaptive functions, and one of the functions of anger is that it can motivate defensive and protective behavior in response to perceived threat [26]. Because people with high punishment sensitivity will be more inclined to experience threat, they might also be more inclined to respond with reactive aggression/anger in ambiguous situations that might be interpreted as threatening [27].

Another pathway may stem from high reward sensitivity. Multiple studies have indicated an association between reward sensitivity and anger problems. More specifically, reward sensitivity has been associated with self-reported conduct problems in clinical adolescents [28], trait anger in non-clinical students [29, 30], self-reported verbal and physical aggression in non-clinical students [29], and self-reported hostility in non-clinical students [30]. People with high reward sensitivity are highly motivated to gain rewards, more responsive to reward, and have more attention to rewarding cues in the environment. Therefore, people with high reward sensitivity have higher reward expectancies in ambiguous situations that may involve potential rewards [12, 31]. This may result in the person taking action to gain the reward [32], and may have beneficial effects in daily life when these rewards are indeed obtained [33]. If people with high reward sensitivity indeed receive their expected rewards, this will lead to positive emotions [34]. However, given their high reward expectancy, they are also more prone to detect non-reward (rewards with a lower than expected frequency or lower level of reward), which will lead to anger out of frustrative non-reward [27, 30, 31, 34, 35]. Anger out of non-reward situations is therefore expected to be heightened in persons with high reward sensitivity [31]. Because people with high reward sensitivity will be more inclined to experience situations as involving non-reward, they will also be more inclined to respond with anger [27]. Following this, high reward sensitivity might set individuals with anxiety disorders at risk to develop comorbid anger problems.

A series of previous studies in adult samples provided evidence for such relationships between punishment sensitivity/reward sensitivity and anger. In these studies, participants had to read a series of scenarios reflecting common anger eliciting situations and to indicate the level of anger that each situation would elicit. Two of these studies were conducted in large undergraduate samples (N = 466 [35]; N = 323 [29]), and one in a non-student adult sample (N = 100 [36]). In these studies, the total anger score across these daily situations was associated with both high punishment and high reward sensitivity.

**Fig. 1:**
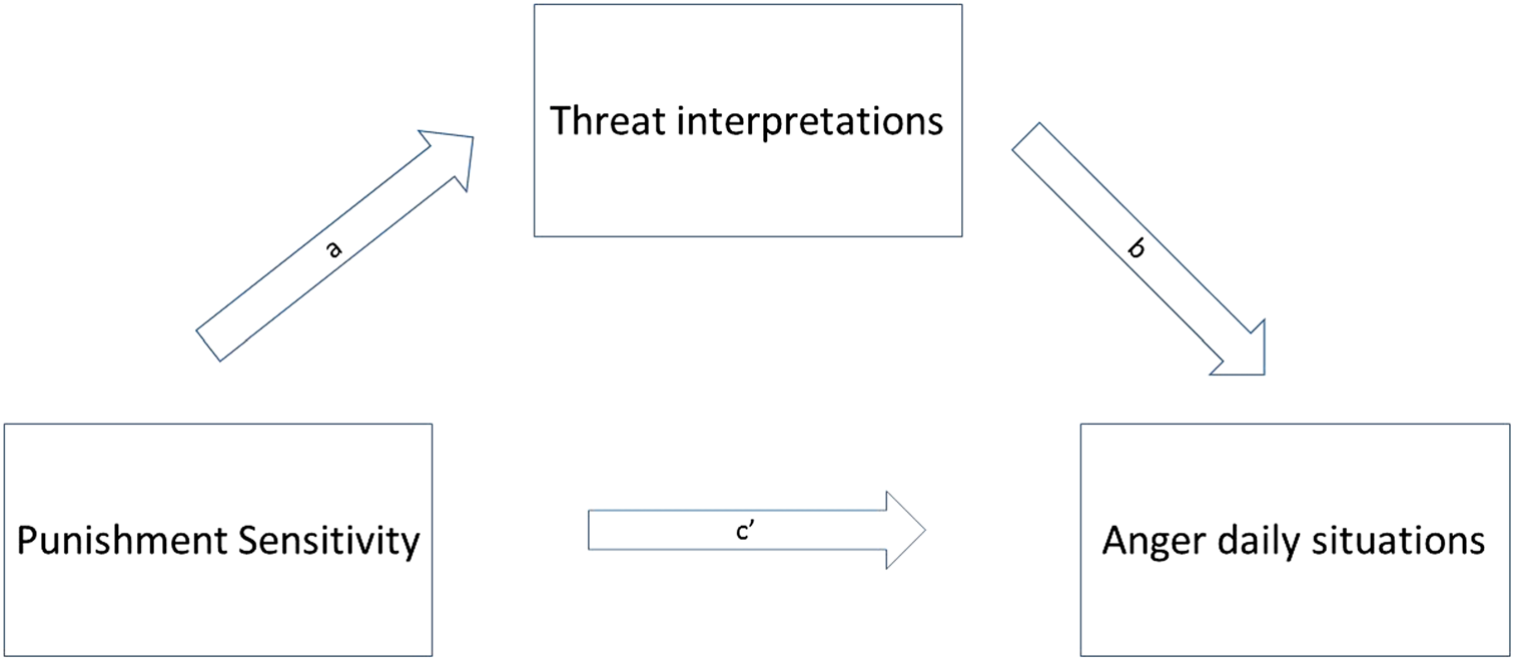
Expected mediation model for the association between punishment sensitivity and anger via threat interpretations

**Fig. 2:**
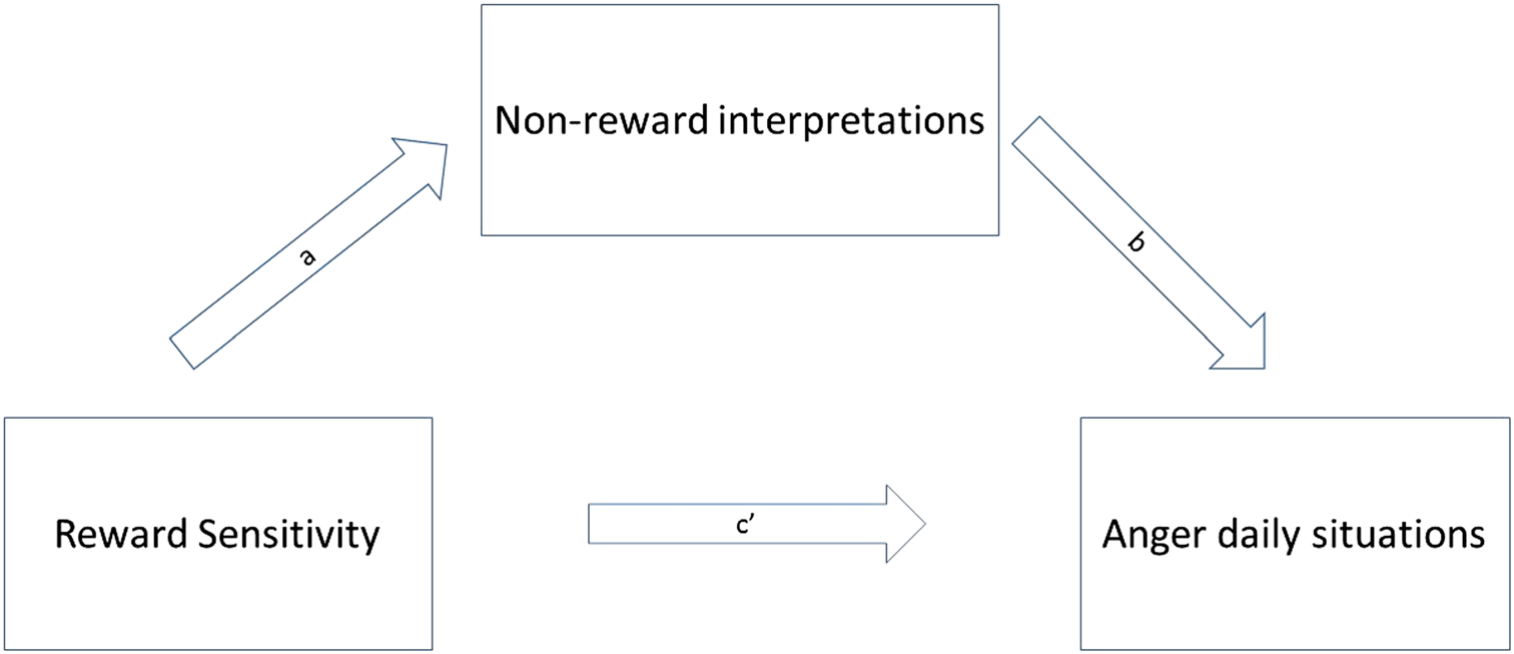
Expected mediation model for the association between reward sensitivity and anger via non-reward interpretations

The current study was designed to investigate whether these relationships are also evident in adolescents and may help explain comorbid anger problems in CYP with anxiety disorders. Punishment and reward sensitivity have been found to increase across adolescence, peaking in young adulthood, and declining into later adulthood. Therefore, in adolescence, we would expect punishment and reward sensitivity to be relatively high compared to later adulthood [37]. This indicates that punishment and reward sensitivity are especially relevant to investigate as factors influencing daily life emotions in the developmental period of adolescence and young adulthood. Also, psychopathology typically rises during adolescence, and research suggests that adult mental health burden largely results from disorders with onset in childhood and adolescence [38]. It is therefore important to test proposed mechanisms to psychopathology not only in adults but also specifically in the age period in which people are vulnerable to develop psychopathology. Furthermore, as an important subsequent step, we also examined the mechanisms that were proposed to underlie these relationships. More specifically, we examined whether indeed high punishment sensitivity would be associated with anger via perceived threat (see Fig. 1), and whether high reward sensitivity would be associated with anger via perceived non-reward (see Fig. 2).

In short, this study focused on the mechanisms that may underlie the relationship between punishment-/reward sensitivity and anger to help improve our understanding of how anger problems develop in adolescents. This is not only of theoretical relevance but can also provide clues for how comorbid behavioral problems in CYP with anxiety disorders might best be targeted in interventions. If these comorbid behavioral problems indeed arise out of threat situations, anxiety focused treatment is expected to be sufficient for also treating the behavioral problems, given that these threat situations are targeted in the treatment. However, if these behavioral problems arise out of non-reward situations, another treatment approach or the addition of modules focusing on the behavioral problems may be necessary.

## Method

### Power Calculation

We calculated the power for the analysis that needed the highest sample size. Using GPower 3.1.9.4, we calculated the power when conducting linear multiple regression including five tested coefficient (a, b, c, c’, ab, see Fig. 1) and where we were interested in the R^2^ increase. We wanted to be able to detect a small to moderate effect size (f^2^ = 0.09), in line with the reported small to medium correlations found in similar studies [29, 35, 36]. For a power of 0.80 and an alpha of 0.05, a total sample size of N = 149 was needed.”

### Participants

Participants were 158 adolescents (56% girl, 99% white), with ages ranging from 15 to 17 years, and a mean age of 15.7 years. We contacted a large high school located in the southern area of the Netherlands and were granted permission to ask the students to participate in our study. Emails were sent around to parents of children from this high school. In total, 354 parents of adolescents were contacted and 234 parents gave permission for their child to participate. Of those, 160 adolescents participated, of which one participant only filled out the CHIA questionnaire and one participant did not finish any questionnaire. Therefore, analyses were conducted with the remaining sample of 158 participants.

## Measurements

### Reward- and Punishment Sensitivity

The Reward and Punishment Responsivity and Motivation Questionnaire (RPRM-Q; [39]) was used to measure aspects of reward and punishment sensitivity. We used this questionnaire, since it measures reward and punishment sensitivity independent of specific stimuli, thereby preventing conceptual overlap with the measures of anger and anxiety. The RPRM-Q consists of 18 items answered on a 5-point scale ranging from [1] “This does not apply to me at all” to [5]“This applies to me completely”. A punishment sensitivity score (Cronbach’s $\alpha$ = 0.85) was computed by summing the items from the punishment responsivity and motivation to avoid punishment subscales; in a similar way a reward sensitivity score (Cronbach’s $\alpha$ = 0.88) was computed by summing the items from the reward responsivity and motivation to approach reward subscales.

### Anger in Daily Situations

The Children’s Inventory of Anger (CHIA; [40] was administered to measure anger in daily situations. The original questionnaire measures anger in 39 descriptions of daily anger-provoking situations on a 4-point scale ranging from [1]*“I don’t care. That situation doesn’t bother me. I don’t know why that would make anyone angry or mad”* to [4];*“I can’t stand that! I’m furious! I feel like really hurting or killing that person, or destroying that thing!”.* The 1-week test–retest reliability is good (r = 0.75; [40]). The questionnaire is originally in English, and was translated into Dutch by using a back and forth translation in collaboration with a native English and Dutch speaker who also holds a bachelor degree in English language. The translation was approved by the publishing organization of the CHIA. A total score for anger in daily situations was calculated by summing the anger scores for the 39 situations per person. The estimate of the reliability in the current study was high (Cronbach’s $\alpha$ = 0.91).

### Interpretation of Threat and Non-Reward in Daily Situations

After the administration of the CHIA for measuring anger, participants again read the daily situations and were asked to indicate how they viewed the situation with regard to level of threat and level of non-reward. They indicated the level of threat and non-reward on a 4-point scale from 1 (not at all), to 4 (a lot). To measure non-reward, the following explanation was given: *The first question is to indicate to what extent you think that in this situation the getting of something nice that you wanted is obstructed/hindered. With obstructed/hindered we mean: to what extent something not succeeded, something was not allowed or you could not get something. Importantly: it is about what you think and feel in this situation. You can choose from: not at all, a bit, quite a lot, a lot.*

To measure threat the following explanation was given: *The second question is to indicate to what extent you think this situation is threatening (not at all threatening to very threatening). Threatening here means physical threat (for example that someone wants to hurt you) but also threat in social situations (for example that you are rejected). Importantly: it is about what you think and feel in this situation. You can choose from: not at all, a bit, quite a lot, a lot.* A total score for threat interpretations in daily situations was calculated by summing the threat scores for the 39 situations per person, in a similar way, a total score for non-reward interpretations in daily situations was calculated. The estimates of the reliability of the threat interpretation scale (Cronbach's α =0.94) and the non-reward interpretation scale (Cronbach's α =0.94) of the CHIA were high in this study.

Furthermore, in order to describe the anxiety levels and behavioral problems of our current sample, participants also completed questionnaire measures of anxiety symptoms and behavioral problems.

### Anxiety

Anxiety symptoms were assessed using the Revised Child Anxiety and Depression scale-Child version (RCADS-C; [41, 42]. The RCADS-C is a self-report questionnaire that originally consisted of 47 items that measures the current occurrence of symptoms of DSM-IV anxiety disorders and depression on a 4-point scale ranging from 0-never to 3-always in children from the ages of 7 to 19. In the current study, we calculated a total anxiety score consisting of only those subscales that correspond to the primary anxiety disorders of children: separation anxiety disorder, social phobia, generalized anxiety disorder, and panic disorder. This is in line with the DSM 5 categorization of anxiety disorders and included 31 items. The estimate of the reliability of this total anxiety scale in the current study was high (Cronbach’s $\alpha$ = 0.95).

### Behavioral Problems

Behavioral problems were assessed with the aggressive behavior subscale of the Youth Self Report (YSR) [43] which consists of 17 items and measures the occurrence of aggressive behaviors over the past 6 months on a 3-point scale ranging from not at all (0) to clearly/often [2] (e.g. ‘I argue a lot, ‘I don’t obey the rules, at home at school or somewhere else’, ‘I get into fights’). The reliability estimate of this aggressive behavior subscale was high (Cronbach’s α = 0.81).

### Procedure

This study used a cross-sectional design and adolescents were recruited from multiple classes in a high school in the Netherlands. Adolescents completed the questionnaires in a classroom during a tutor class via the online questionnaire platform Qualtrics. It took about 45 min to complete all questions. They first filled in some demographics, followed by the daily situations with ratings on anger and anxiety (because the anxiety ratings are not relevant for the current research questions they are not included in this study), the daily situations with ratings on non-reward and threat, a questionnaire on punishment and reward sensitivity, and lastly questionnaires on anxiety symptoms and behavioral problems.

### Analytic Plan

(i) We first tested whether both punishment- and reward sensitivity predicted anger responses in daily situations. We expected that both punishment- and reward sensitivity explain unique variance in the total anger score. We therefore conducted a hierarchical regression analysis including both punishment- and reward sensitivity as predictors and total anger as dependent variable. Reward sensitivity was included in step 1; punishment sensitivity was added in step 2.
(ii) If we would find a significant association between punishment sensitivity and total anger over situations, we planned to conduct an examination of indirect effects, where our dependent variable (y) was the total anger score, our independent variable (x) was punishment sensitivity, and our mediator (m) was the total threat interpretation score. Similarly, if we would find an association between reward sensitivity and total anger over situations, we planned to conduct an examination of indirect effects, where our dependent (y) variable was the total anger score, our independent variable (x) was reward sensitivity, and our mediator (m) was the total non-reward interpretation score. These analyses would be conducted in SPSS using Hayes model 4 from Process version 3.4.
(iii) Lastly, we planned to assess the specificity of these pathways, by testing whether reward sensitivity was also associated with total anger via threat interpretations, and punishment sensitivity with total anger via non-reward interpretations, by conducting analysis examining indirect effects using Hayes model 4.

## Results

### Descriptives

With regard to anxiety symptoms, the mean item score on the RCADS was 0.58 (SD = 0.41, range 0.03–2.29), indicating that the mean score of this sample falls within the non-clinical range [44]. For behavioral problems, the mean item score on the YSR aggressive behavior scale was 0.32 (SD = 0.33, range = 0–2), also falling in the non-clinical range [43]. Table 1 presents the mean item scores, standard deviations and inter-correlations for all variables included in the analyses.

**Table 1:** Means, standard deviations, and inter-correlations for variables included in the analyses

Variables	Mean item score	SD	Min–max	1	2	3	4
1. Punishment sensitivity	3.04	0.71	1.33–4.78	−			
2. Reward sensitivity	3.72	0.55	2.00–4.89	0.21*	−		
3. Threat interpretation	1.43	0.38	1.00–2.85	0.39*	0.11	−	
4. Non-reward interpretation	2.29	0.48	1.00–3.85	0.23*	0.24*	0.42*	−
5. Total anger	2.13	0.36	1.10–3.31	0.22*	0.24*	0.37*	0.67*

*Significant with α = 0.05

Inspection of regression residuals for the regression and analyses examining indirect effects revealed no violations of assumptions (linearity, homoscedasticity, normality, see Appendix A).

#### Analysis i: Do punishment- and reward sensitivity predict anger responses in daily situations?

We first tested whether both punishment- and reward sensitivity showed independent associations with anger responses in daily situations by conducting a hierarchical regression analysis. A significant positive association was found between reward sensitivity and total anger score in step 1 (see Table 2). When punishment sensitivity was added in step 2, we found that punishment sensitivity was also significantly and positively associated with total anger, the association of reward sensitivity with anger also remained significant in step 2. Furthermore, adding punishment sensitivity to the regression analysis resulted in a significant increase in explained variance of total anger (see Table 2).

**Table 2:** Hierarchical regression model with total anger score as dependent variable and punishment-and reward sensitivity as predictors

		*b*	SE *b*	*Beta*	*t*	*p*
Step 1	Constant	60.45	7.41		8.16	< 0.001
	Reward sensitivity	0.67	0.22	.24	3.07	0.003*
Step 2	Constant	53.49	7.96		6.72	< 0.001
	Reward sensitivity	0.57	0.22	0.20	2.56	0.011*
	Punishment sensitivity	0.38	0.17	0.17	2.22	0.028*

*R*^*2*^_*change.* =_.057 *F* = 9.41 (1.156) *p* = .003

*R*^*2*^_*change* =_.029 *F* = 4.91 (1.155) *p* = .028

^*^Significant with *α* = .05

#### Analysis ii: Do threat interpretations mediate the association between punishment sensitivity and anger, and do non-reward interpretations mediate the association between reward sensitivity and anger?

**Fig. 3:**
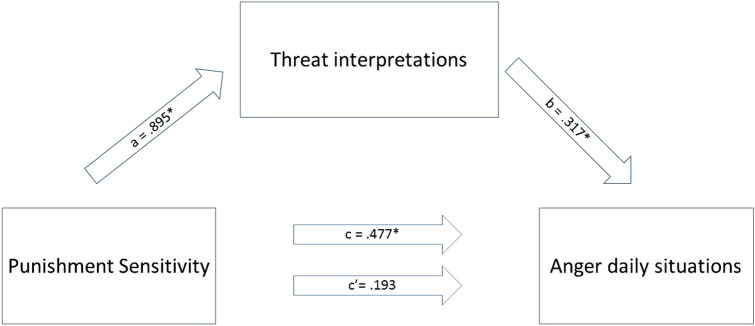
Mediation model for the association between punishment sensitivity and anger via threat interpretations

A simple examination of indirect effects was conducted to test whether the relationship between punishment sensitivity and total anger can be accounted for by threat interpretations. In Table 3 the results of this analysis are presented. Regressing punishment sensitivity on total anger indicated a significant total effect (path *c*), which became insignificant in the full model (direct effect; path *c′*). Additionally, punishment sensitivity significantly predicted threat interpretations (path *a*), and the strength of threat interpretations was a significant predictor of anger, path *b*). The indirect effect of punishment sensitivity on anger (path *ab*) showed a confidence interval that did not include zero, indicating an indirect effect of punishment sensitivity on anger via perceived threat (see also Fig. 3).

**Table 3:** Mediation model including total anger as dependent variable, punishment sensitivity as independent variable and threat interpretations as mediator

	Path/effect	*B*	SE	*t*	*p*	95% CI
Simple regression models						
*R*^*2*^ = *.05 F* (1,156) = 7.71 *p* = .006	*c* (total effect of PS on anger)	0.477	0.172	2.78	0.006*	0.14;0.82
*R*^*2*^ = *.15 F* (1,156) = 27.70 *p* < .001	*a* (PS on threat interpretations)	0.895	0.170	5.26	< 0.001*	0.56;1.23
Multiple regression model*						
*R*^*2*^ = *.14 F* (2,155) = 12.71 *p* < .001	*c′*(direct effect of PS on anger)	0.193	0.178	1.09	0.278	− 0.16; 0.54
	*b* (threat interpretations on anger)	0.317	0.077	4.11	< 0.001*	0.17; 0.47

		Effect	Boot SE			Boot CI
	*ab* (indirect effect of PS on anger through threat interpretations)	0.284	0.078			0.14; 0.45

PS = punishment sensitivity

Anger = total anger score over daily situations

Boot = bootstrap

*Significant with *α* of 0.05

**Fig. 4:**
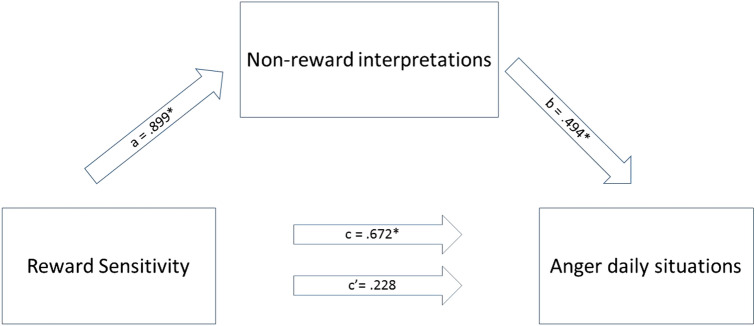
Mediation model for the association between reward sensitivity and anger via non-reward interpretations

In a similar way, an examination of indirect effects was conducted to test whether the relationship between reward sensitivity and total anger can be accounted for by non-reward interpretations. We found an indirect effect of reward sensitivity on anger via perceived non-reward (see Table 4 and Fig. 4).

**Table 4:** Mediation model including total anger as dependent variable, reward sensitivity as independent variable and non-reward interpretations as mediator

	Path/effect	*B*	SE	*t*	*p*	95% CI
Simple regression models						
*R*^*2*^ = *.06 F* (1,156*)* = 9.41 *p* = .003	*c* (total effect of RS on anger)	0.672	0.219	3.07	0.0023*	0.24;1.11
*R*^*2*^ = *.06 F* (1,156) = 9.57 *p* = .002	*a* (RS on non-reward interpretations)	0.899	0.291	3.09	< 0.002*	0.33;1.47
Multiple Regression Model*						
*R*^*2*^ = *.46 F* (2,155) = 66.30 *p* < .001	*c′*(direct effect of RS on anger)	0.228	0.171	1.33	0.184	− 0.11;0.57
	*b* (non-reward interpretations on anger)	0.494	0.046	10.78	< 0.001*	0.40;0.58

		Effect	Boot SE			Boot CI
	*ab* (indirect effect of PS on anger through non-reward interpretations)	0.444	0.161			0.15;0.78

RS = reward Sensitivity

Anger = total anger score over daily situations

Boot = bootstrap

*Significant with *α* of 0.05

#### Analyses iii

Following, we tested the specificity of these pathways, by investigating whether reward sensitivity was also associated with total anger via threat interpretations, and punishment sensitivity with total anger via non-reward interpretations. We found an indirect effect of punishment sensitivity on total anger via non-reward interpretations (see Table 5), but no indirect effect of reward sensitivity on total anger via perceived threat (see Table 6).

**Table 5:** Mediation model including total anger as dependent variable, punishment sensitivity as independent variable and non-reward interpretations as mediator

	Path/effect	*B*	SE	*t*	*p*	95% CI
Simple regression models						
*R*^*2*^ = *.05 F* (1,156) = 7.71 *p* < .006	*c* (total effect of PS on anger)	0.477	0.172	2.78	0.006*	0.14;0.82
*R*^*2*^ = *.05 F* (1,156) = 8.43 *p* = .004	*a* (PS on non-reward interpretations)	0.660	0.227	2.90	0.004*	0.21;1.11
Multiple Regression Model*						
*R*^*2*^ = *.46 F* (2,155) = 65.82 *p* < .001	*c′*(direct effect of PS on anger)	0.149	0.133	1.12	0.265	− 0.11;.0.41
	*b* (non-reward interpretations on anger)	0.497	0.046	10.87	< 0.001*	0.41;0.59

		Effect	Boot SE			Boot CI
	*ab* (indirect effect of PS on anger through non-reward interpretations)	0.328	0.129			0.08;0.59

PS = punishment sensitivity

Anger = total anger score over daily situations

Boot = bootstrap

^*^Significant with *α* of .05

**Table 6:** Mediation model including total anger as dependent variable, reward sensitivity as independent variable and Threat interpretations as mediator

	Path/effect	*B*	SE	*t*	*p*	95% CI
Simple regression models						
*R*^*2*^ = *.06 F* (1,156) = 9.41 *p* < .003	*c* (total effect of RS on anger)	0.672	0.219	3.07	0.003*	0.24;1.11
*R*^*2*^ = *.01 F* (1,156) = 1.73 *p* = .190	*a* (RS on threat interpretations)	0.309	0.235	1.32	0.190	− 0.16;0.77
Multiple Regression Model*						
*R*^*2*^ = *.18 F* (2,155) = 16.41 *p* < .001	*c′*(direct effect of RS on anger)	0.570	0.207	2.76	0.007*	0.16;0.98
	*b* (threat interpretations on anger)	0.329	0.070	4.71	< 0.001*	0.19;0.47

		Effect	Boot SE			Boot CI
	*ab* (indirect effect of RS on anger through threat interpretations)	0.102	0.079			− 0.04;0.27

RS = reward sensitivity

Anger = total anger score over daily situations

Boot = bootstrap

*Significant with *α* of 0.05

## Discussion

### Summary of Main Results

In line with previous studies in adult samples [29, 35, 36], we found higher anger responses in adolescents with relatively high reward and/or relatively high punishment sensitivity. Both punishment and reward sensitivity showed partly independent associations with anger responses in daily situations in adolescents**.** As an important next step, we also examined the mechanisms that were proposed to underlie these relationships. In line with our hypotheses, we found an indirect effect of punishment sensitivity on anger via perceived threat, and an indirect effect of reward sensitivity on anger via perceived non-reward. We additionally found an indirect effect of punishment sensitivity on anger via perceived non-reward.

### Evidence for the Two Candidate Pathways towards Anger

This study was designed to test two candidate pathways that may help explain anger responses in anxious youth. Given that psychopathology typically rises during adolescence and research suggests that adult mental health burden largely results from disorders with onset in childhood and adolescence [38], it is important to test proposed mechanisms to psychopathology not only in adults but also specifically in the age period in which people are vulnerable to develop psychopathology. Following the first pathway, it was expected that people with high punishment sensitivity would be more inclined to experience threat, and therefore also be more inclined to respond with anger. Following the second pathway, it was expected that people with high reward sensitivity would be more inclined to experience non-reward, and would therefore also be more inclined to respond with anger. In the current study we found evidence for these pathways in a non-clinical adolescent sample. The finding of an indirect effect of punishment sensitivity on anger via perceived threat is consistent with the idea that people with high punishment sensitivity are more inclined to experience threat and might therefore be also more inclined to respond with reactive aggression/anger in ambiguous situations. This finding corresponds with the adaptive function of anger to motivate defensive and protective behavior in response to perceived threat. There is ample evidence indicating that children and adults with anxiety symptoms and anxiety disorders are characterized by high punishment sensitivity [19–22]. Therefore, this pathway, stemming from high punishment sensitivity, might be especially relevant to explain comorbid anger problems in adolescents with anxiety disorders. Additionally, the finding of an indirect effect of reward sensitivity on anger via perceived non-reward is consistent with the idea that individuals with higher reward sensitivity are also more sensitive for non-reward, which then increases the chance for responding with anger in ambiguous situations. Following this, adolescents with anxiety disorders that are also high reward sensitive, might have a relatively higher chance to be also vulnerable to develop comorbid anger problems.

Also an indirect effect of punishment sensitivity on anger was found via non-reward interpretations. The constructs of punishment and reward sensitivity are derived from the reinforcement sensitivity theory [12]. In the original theory, reward sensitivity mediates reactions to appetitive stimuli (i.e., reward and termination/omission of punishment) whereas punishment sensitivity is proposed to mediate reactions to aversive stimuli (i.e., punishment and the omission/termination of reward). Our finding that punishment sensitivity is also associated with non-reward interpretations is in line with this original theory. However, our current findings also clearly indicate an association of reward sensitivity with non-reward interpretations. This finding is more in line with recent commentaries on the predictions of the reinforcement sensitivity theory with regard to associations of punishment and reward sensitivity with frustrative non-reward, highlighting the importance of people’s expectancies. People with high reward sensitivity have higher reward expectancies in ambiguous situations that may involve potential rewards [12, 31]. Given their high reward expectancy, they are also prone to detect non-reward (rewards with a lower than expected frequency or lower level of reward), which will lead to anger out of frustrative non-reward [27, 30, 31, 34, 35]. Therefore, both reward and punishment sensitivity might play a role in making non-reward interpretations, which is in line with our current findings. Building further on our findings, a next step would be to further investigate the differential role of punishment and reward sensitivity in predicting anger. This could be done by setting up a similar study in a larger sample where more complex models could be tested, including both punishment and reward sensitivity as competing predictors of anger, with non-reward and threat interpretations as variables explaining these indirect effects.

### Clinical Implications

An important next step would be to investigate whether these pathways to anger can also be found in a clinical sample. It should then be examined whether indeed adolescents showing both clinical levels of anxiety and comorbid behavioral problems are characterized by relatively high levels of punishment sensitivity and/or reward sensitivity. To the extent that behavioral problems are due to experienced threat (e.a. following the threat pathway), they are expected to improve following anxiety focused CBT, since this treatment reduces experienced threat in situations. In line with this, a recent meta-analysis showed that behavioral problems in children and adolescents generally tend to reduce following anxiety-focused CBT [45]. However, for children for whom this is not the case, and where anger might also arise out of non-reward situations (which could stem from high reward and/or high punishment sensitivity), more specific attention should probably be paid to these problems in treatment [46, 47], given that anxiety-focused CBT does not specifically target these kind of situations. Weisz et al. [47] developed a modular approach, including modules focusing on anxiety, depression, and behavioral problems that can be flexibly applied by a therapist based on weekly scores on different symptomatology and progress. Quicker gains were reported for children treated with this flexible approach than for children treated with the standard evidence-based treatment. Furthermore, the modular approach seemed to have most advantage after 1-year follow-up [48]. These findings might indicate that for children presenting with behavioral problems out of non-reward, it might be beneficial to provide treatment with modules that specifically focus on behavioral problems.

### Limitations and Recommendations for Future Research

Our study indicates that individual differences in punishment- and reward sensitivity might help in understanding anger responses in adolescents. The current study used standardized hypothetical situations where people indicated how angry they would become using self-report scales. However, whether these kind of situations are also experienced in the daily lives of adolescents and play a role in triggering anger in daily life remains to be tested. Experience sampling methods might be helpful in this regard, where data is collected in daily life and the relevance of the proposed pathways can be tested in individually meaningful real life situations. A study using this methodology already investigated associations of punishment and reward sensitivity with emotions in daily life of students and found that both punishment and reward sensitivity were associated with experienced anger in daily life [34]. It would be interesting to complement this earlier study with a study testing whether indeed situations that are experienced as threatening or as involving non-reward also tend to elicit anger responses.

Additionally, the level of experienced threat is expected to play a role in the individuals’ response to threat. Low levels of threat will mainly lead to a freeze response, whereas higher levels of immediate threat will lead to a flight response, and high levels of immediate threat where avoidance of the threat is not possible will lead to reactive aggression [49, 50]. In the current study we tested the influence of perceived threat, but did not specifically focus on threat situations where avoidance was hindered. This could be more specifically investigated in future studies. Furthermore, in this study we used an analysis examining indirect effects using cross-sectional data, which might lead to biased estimates [51]. However, we expect that in daily life the time lag between the predicted processes (interpretation of the situation and subsequent anger) is short, and would therefore not be captured accurately in longitudinal designs. However, experimental designs in which individuals are exposed to situations in which levels of non-reward and threat are experimentally manipulated might help in investigating whether individual differences in punishment- and reward sensitivity are related to differential effects of these different conditions on anger. A way to take the temporal order of the processes more into account might be by using ESM designs where participants with varying levels of punishment –and reward sensitivity fill out daily diaries on situations where they indicate how much they experienced the situations as involving non-reward and/or threat and indicate how angry they felt at the end of the situations. Given the short expected time lag between the processes of interest in the current study, we think the current design provides an appropriate and helpful first step in investigating the potential of these pathways to anger. Importantly, the sample of adolescents in our study is reflective of the high school population in this area of the Netherlands. However, given that this sample is predominantly white, it remains to be tested whether the current results also hold in other populations including minority groups and adolescents in non-western countries.

## Summary

In the current study we tested how individual differences in punishment and reward sensitivity might be involved in anger responses to common anger eliciting situations in adolescents, and what mechanism might link these traits and adolescents’ anger responses. It was found that punishment sensitivity was associated with higher anger responses via perceived threat and perceived non-reward, and reward sensitivity was associated with higher anger responses via perceived non-reward. These pathways may help explain how comorbid anger problems might arise in anxiety disordered children and might provide clues for improving treatment.

## Supplementary Information

Below is the link to the electronic supplementary material.

Supplementary file1 (DOCX 412 kb)
