# Supplementary material for: Anger Responses in Adolescents: Relationship with Punishment and Reward Sensitivity
Source: Child Psychiatry Hum Dev. 2021 Jun 7;53(6):1174–85. doi: 10.1007/s10578-021-01191-w (PMC9561000; doi:10.1007/s10578-021-01191-w)

**Appendix A**

Assumptions check analysis i (regression model including RS, PS and total anger)


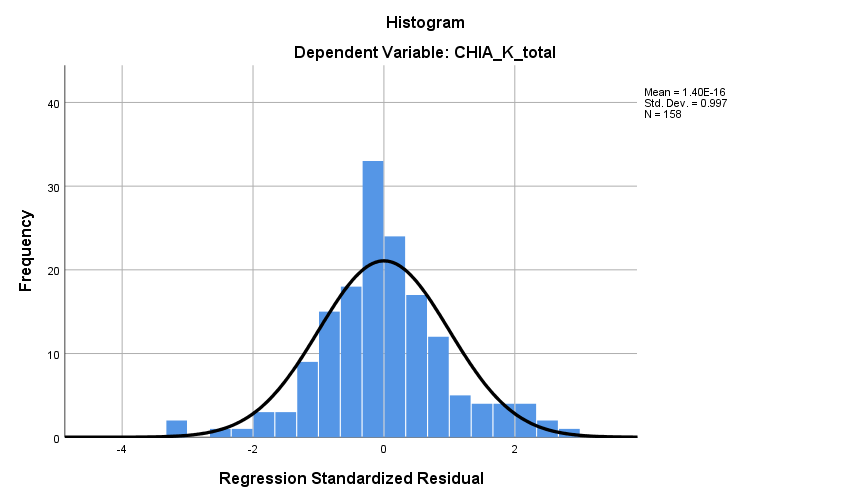


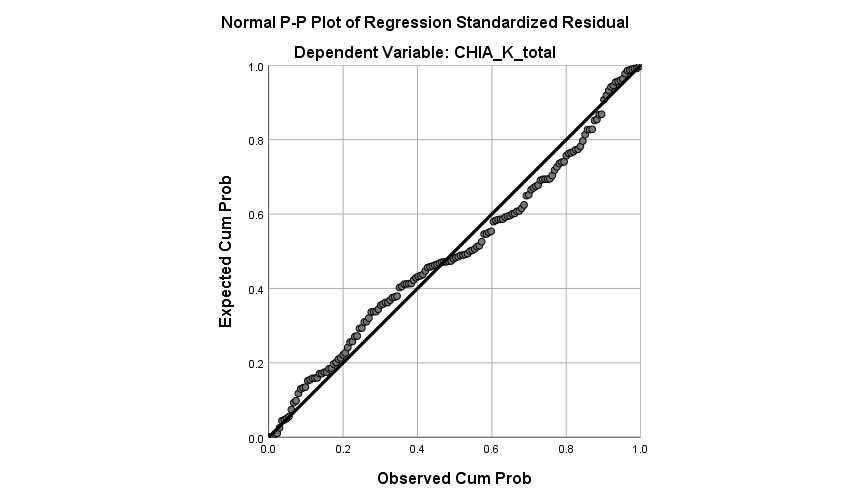


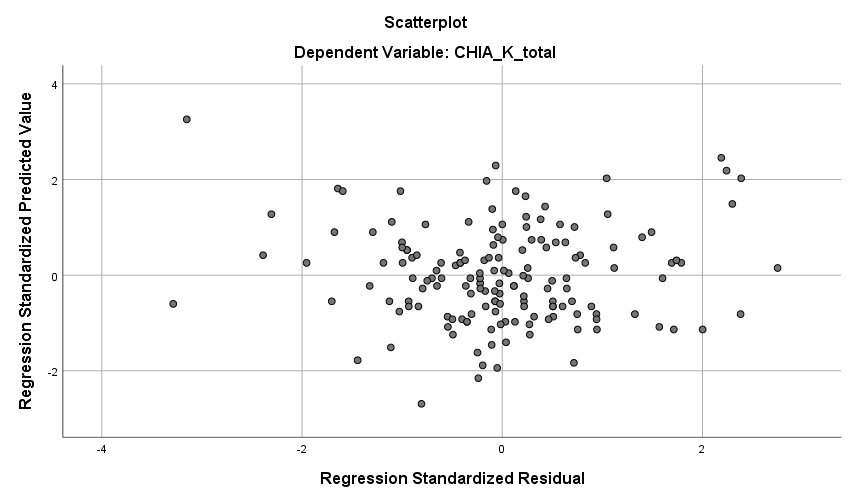


Assumptions check analysis ii (mediation model including PS, threat interpretations, and total anger)


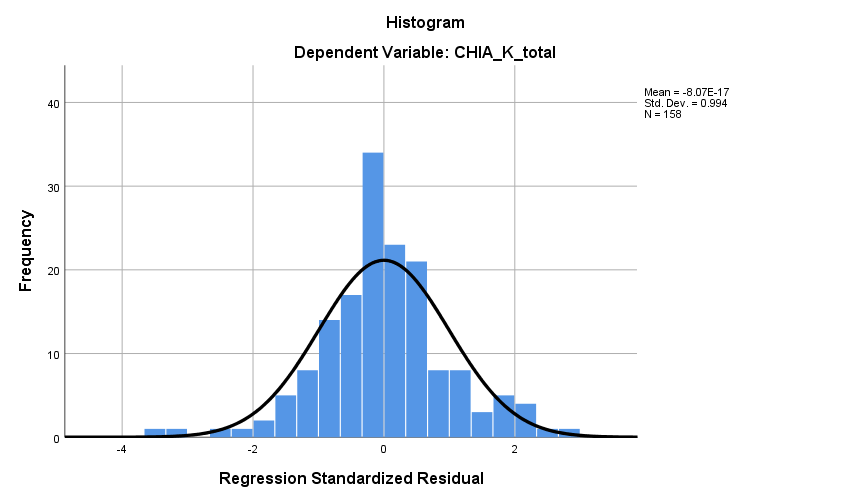


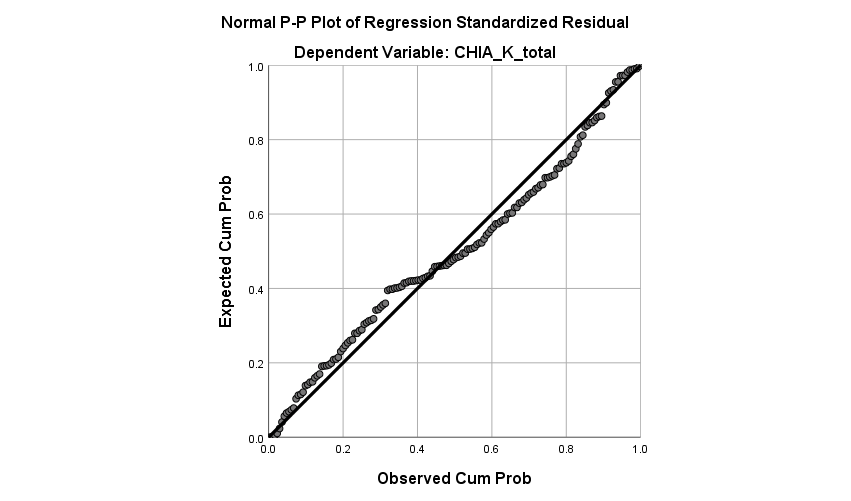


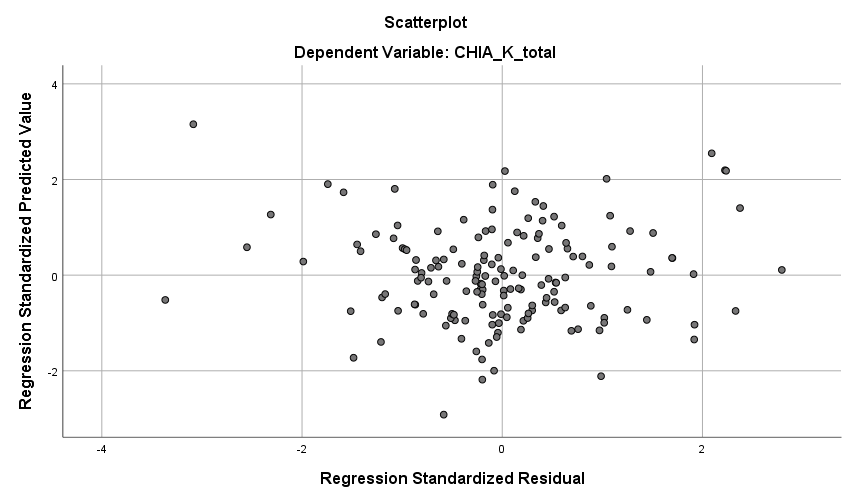


Assumptions check analysis ii (mediation model including RS, non-reward interpretations, and total anger)


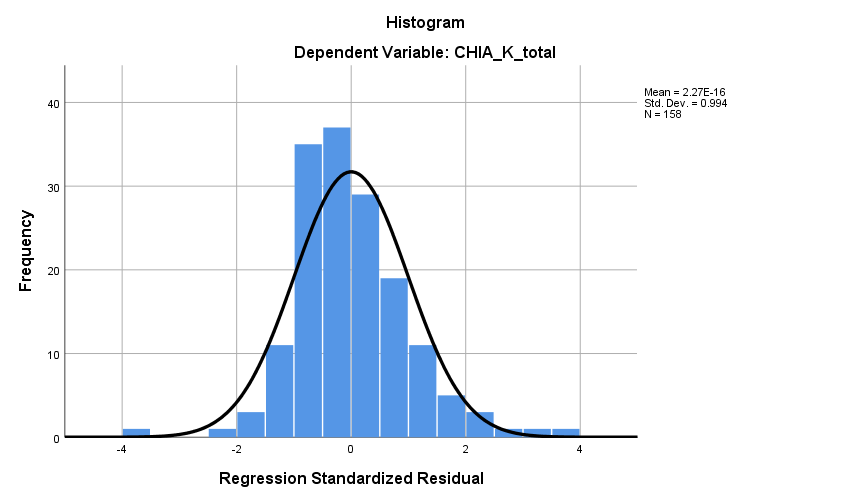


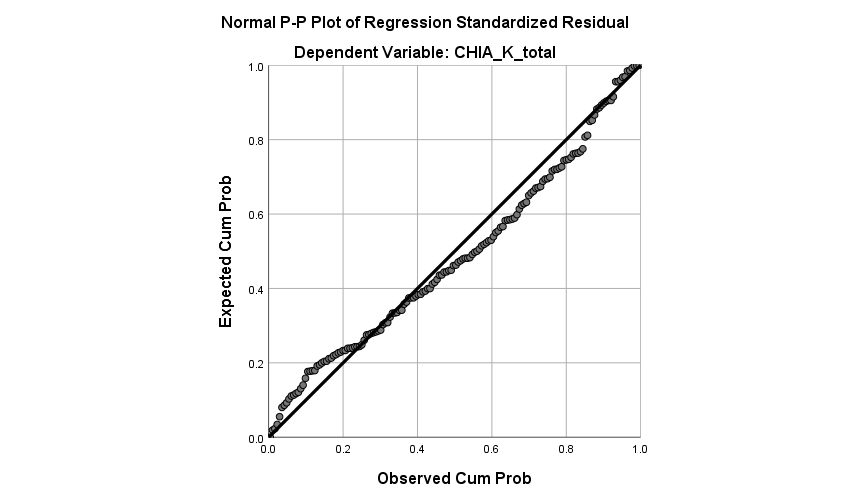


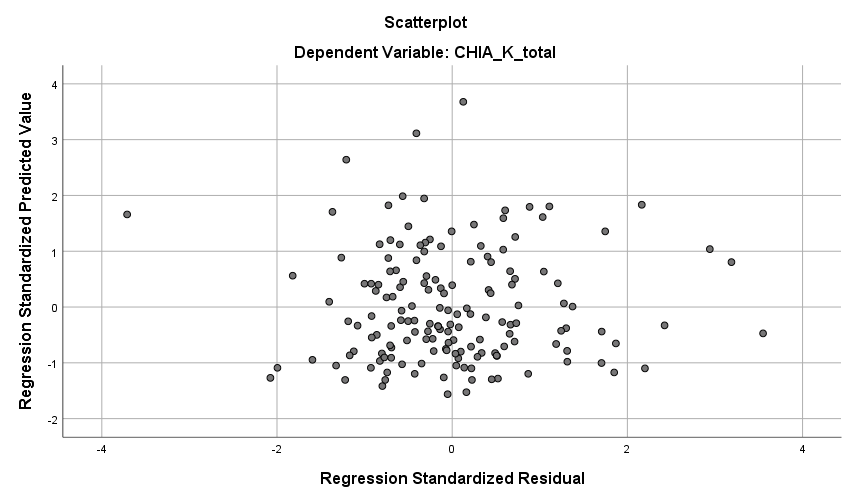


Assumptions check analysis iii (mediation model including PS, non-reward interpretations, and total anger)


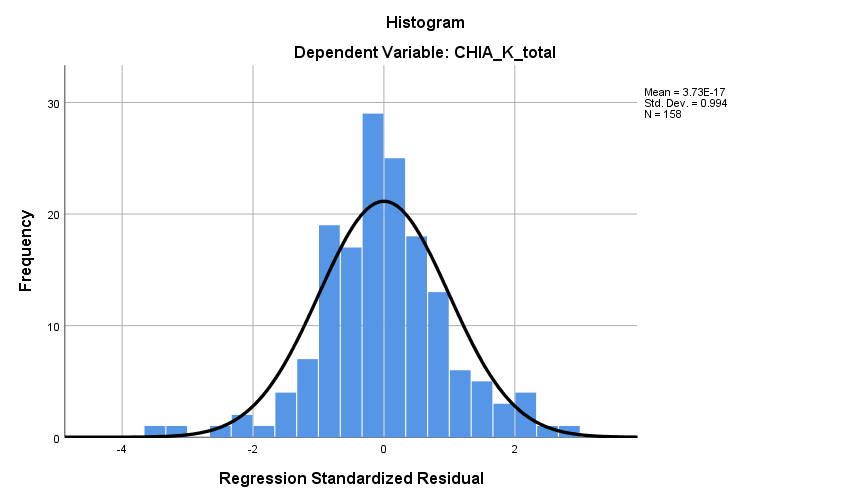


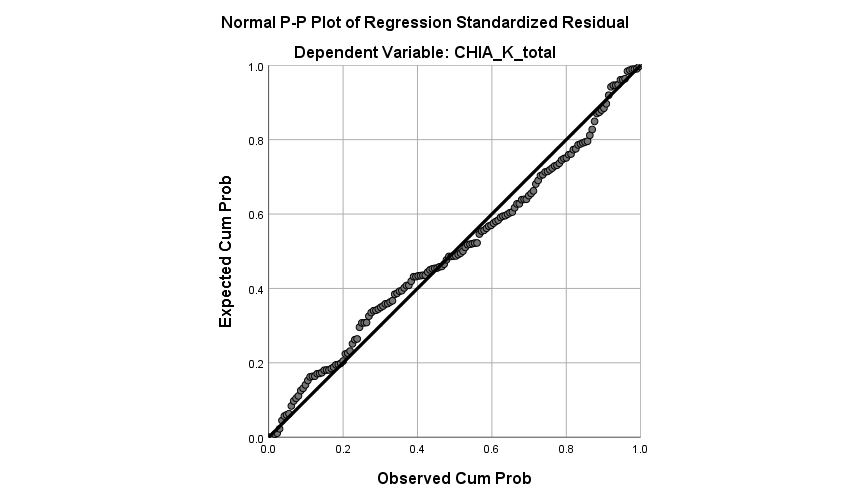


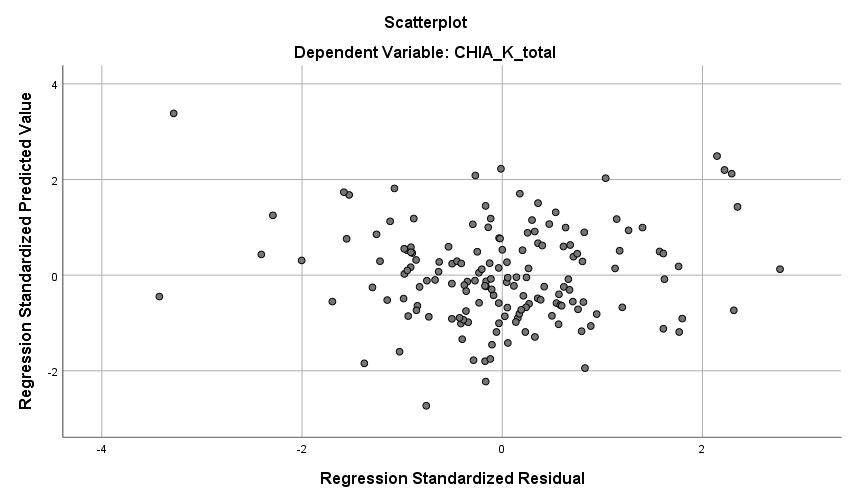


Assumptions check analysis iii (mediation model including RS, threat interpretations, and total anger)


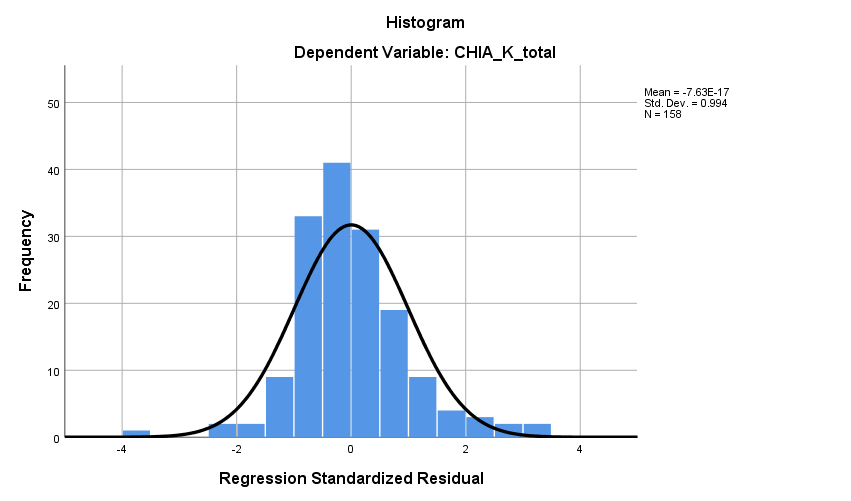


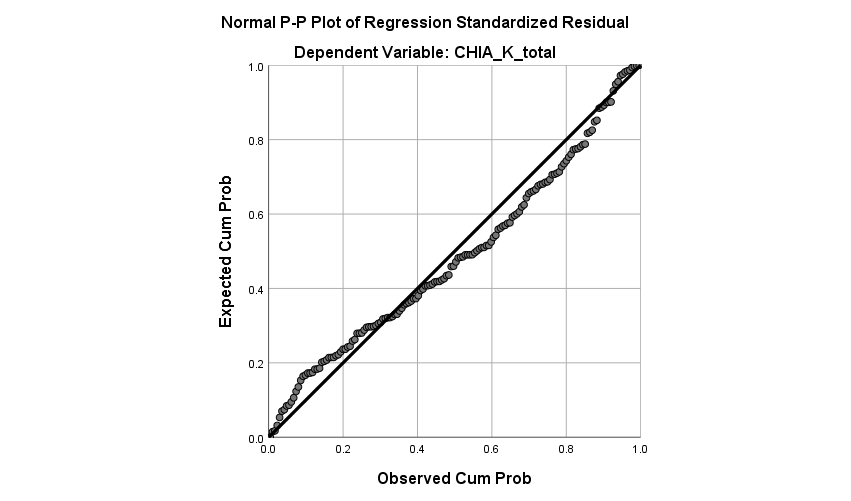


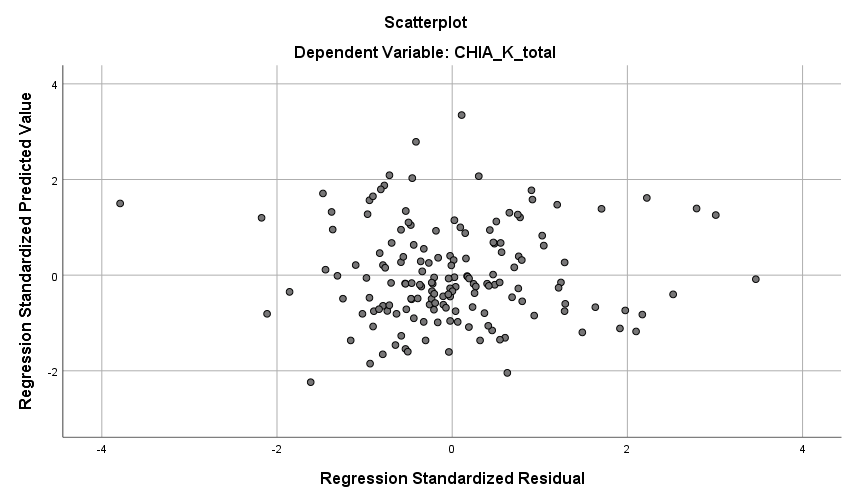

Supplement: Supplementary file 1 — Supplementary file1 (DOCX 412 kb) [file 10578_2021_1191_MOESM1_ESM.docx]
